# Diagnostic accuracy of metastatic axillary lymph nodes in breast MRI

**DOI:** 10.1186/s40064-016-2419-7

**Published:** 2016-06-16

**Authors:** Gozde Arslan, Kubra Murzoglu Altintoprak, Inci Kizildag Yirgin, Mehmet Mahir Atasoy, Levent Celik

**Affiliations:** Department of Radiology, Maltepe University Hospital, Maltepe University, Feyzullah Caddesi, Istanbul, Turkey; Department of Radiology, Sakarya Hendek State Hospital, Sakarya, Turkey; Department of Radiological Imaging, Maltepe University, Istanbul, Turkey

**Keywords:** Axilla, MRI, Lymph node

## Abstract

**Purpose:**

The most important prognostic variable for early stage breast cancer is the status of axillary lymph nodes. The aim of this study was to evaluate the diagnostic accuracy of preoperative magnetic resonance imaging (MRI) for metastatic axillary lymph node in breast cancer cases with post-operative sentinel lymph node biopsy (SLNB) results.

**Materials and methods:**

Women aged between 21 and 73 years who were diagnosed with malignant mass lesion of the breast between 2013 and 2015 were included in this study. The preoperative MR images of patients with diagnosis of breast cancer was evaluated to determine axillary lymph node status. Axillary lymph node size, long axis to short axis ratio, lymph node contours, cortical thickness to anteroposterior diameter ratio, the presence of a fatty hilum and contrast enhancement patterns (homogenous or heterogenous) was noted. Additionally, the presence of comet tail sign which a tail extending from an enhancing breast lesion into the parenchyma and might represent ductal infiltration on post-contrast series was also noted. All data obtained from this evaluation was compared with postoperative SLNB results.

**Results:**

Metastatic nodes were found to have a longer short axis when compared to reactive nodes (p = 0.042; p < 0.05). The long axis to short axis ratio was notably lower in metastatic nodes when compared to reactive nodes. Cortical thickness was higher in metastatic nodes when compared to reactive nodes (p = 0.024; p < 0.05). Comet sign was observed in 15 of metastatic nodes (73.3 %) and in one (5 %) reactive node. This difference was statistically significant (p = 0.001; p < 0.01). While fatty hilum was seen in 40 % of metastatic nodes (n = 6), it was seen in all (n = 20) reactive nodes. This difference was statistically significant (p = 0.001; p < 0.01).

**Conclusions:**

MRI is a non invasive sensitive and specific imaging modality for evaluating the axilla. We have shown that with the help of comet tail sign and status of fatty hilum contrast enhanced MRI has the highest sensitivity of 84.7 % for detecting axillary lymph node metastases (Singletary et al. in Semin Surg Oncol 21(1):53–59, 2003).

## Background

Breast cancer is the most commonly observed malignancy in women (Siegel et al. 2012). Magnetic resonance imaging (MRI) can evaluate the breasts and breast cancer related areas such as axillary lymph nodes, skin or pectoral muscle that are important for prognosis. Prognostic factors for breast cancer are axillary lymph node status, tumour diameter, tumour type/grade, lymphatic and vascular invasion, proliferation markers, ethnicity and patients’ age at diagnosis (Cianfrocca and Goldstein 2004). The most important prognostic variable for early stage breast cancer is the status of axillary lymph nodes. There is a correlation between the number of involved axillary lymph nodes and the risk of distant recurrence (Saez et al. 1989; Nemoto et al. 1983).

The status of lymph nodes in patients with breast cancer used to be evaluated with axillary lymph node dissection (ALND). During the last 15 years. Sentinel lymph node biopsy (SLNB) has replaced ALND for grading of patients with clinically lymph node negative breast cancer. Despite being a less invasive method when compared to ALND, SLNB leads to complications such as lymphedema, seroma, localised swelling, pain, paraesthesia, decrease in arm strength, infectious neuropathy and shoulder stiffness in 20 % of patients (Giuliano et al. 2000). 60–70 % of newly diagnosed patients have negative lymph nodes (Kell and Kerin 2004). Therefore this group of patients do not benefit from SLNB, yet are put under the short and long term morbidity risks of this procedure (Cady et al. 1996). This morbidity may be eliminated through the use of non-invasive techniques. However the detection of small metastasis with these methods may be difficult. The American Joint Committee on Cancer (AJCC) defines micrometastasis (pNmi) as metastatic lymph nodes with a maximum diameter of 0.2–2 mm and isolated tumour cells (ITCs) [pN0(i+)] as a lesion of tumour cells smaller than 0.2 mm in diameter or small cell clusters (Singletary et al. 2003). Micrometastasis and isolated tumour cells do not effect general survival. Therefore, the sensitivity for their detection is of less importance (Maaskant-Braat et al. 2011).

In recent years non-invasive methods such as ultrasonography (US) and positron emission tomography–computed tomography (PET–CT) have been suggested for the evaluation of the axilla. MR imaging has several advantages over other imaging modalities such as not utilizing ionizing radiation (when compared to PET) and having low intra and interobserver differences (when compared to US).

The aim of this study was to evaluate the diagnostic accuracy of preoperative conventional magnetic resonance imaging (MRI) for metastatic axillary lymph node in breast cancer cases with post-operative sentinel lymph node biopsy (SLNB) results.

## Methods

This prospective study was conducted at a tertiary university hospital. 35 women aged between 21 and 73 years who were diagnosed with malignant mass lesion of the breast between 2013 and 2015 were included in this study. Institutional ethics committee approved the study and signed informed consent was obtained from all participants.

### MR imaging method

The axillas were scanned by a 1.5-T MR scanner (Intera. Philips Medical Systems. Best. The Netherlands) using a dedicated double-breast surface coil with the patient in the prone position. An axial three-dimensional high-resolution T1-weighted fast gradient echo fat-suppressed sequence [TE/TR 2.4/4.6 ms; inversion delay spectral presaturation attenuated by inversion recovery (SPAIR) 90 ms; flip angle 10°; FOV 360 × 360 × 132 mm^3^ acquired voxel size 0.9 × 0.9 × 2.5 mm^3^ reconstructed voxel size 0.83 × 0.83 × 2.50 mm^3^. Total acquisition time 60 s] was performed before administration of contrast agent, followed by repeat performance of this same sequence at 0, 1, 2, 3, 4, 5 and 7 min after administration of contrast agent. An additional axial T2-weighted fat suppressed spin echo sequence (TE/TR 110/7548 ms; inversion delay SPAIR 80 ms; flip angle 90°; FOV 380 × 380 × 155 mm^3^, acquired voxel size 1.06 × 1.74 × 3.0 mm^3^, reconstructed voxel size 0.94 × 0.94 × 3.00 mm^3^, total acquisition time 242 s) was performed before administration of contrast material. Postcontrast three-dimensional T1-weighted fast gradient-echo dynamic MR images were acquired after administration of 0.1 mmol/kg gadolinium diethylenetriaminepentaacetic acid (Gd-DTPA).

**Fig. 1:**
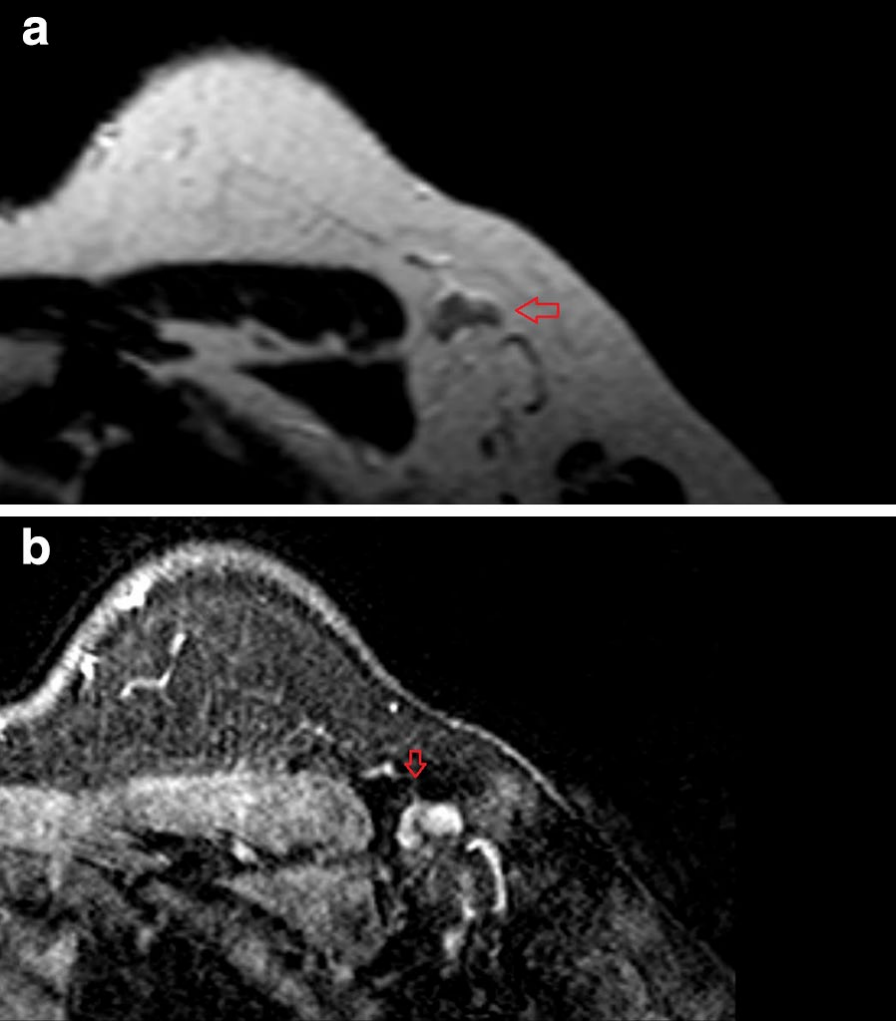
**a** Axial T2WS-TSE MRI showing an enlarged lymph node on the right axilla. Fatty hilum is absent with an increased asymmetrical cortical thickness. **b** The lymph node has a small tail on axial post contrast T1WS MRI, this is called “comet tail sign”

The preoperative MR images of patients with diagnosis of breast cancer was evaluated to determine axillary lymph node status. Axillary lymph node size, long axis to short axis ratio, cortical thickness to anteroposterior (AP) diameter ratio, the presence of a fatty hilum (Fig. 1a) and contrast enhancing patterns (homogenous or heterogenous) on postcontrast series was noted. Additionally, the presence of a comet tail sign on post-contrast series was also noted (Fig. 1b). Comet tail sign was first described for breast lesions by Kaiser (2008). It is a tail extending from an enhancing breast lesion into the parenchyma. The tail is usually directed towards nipple or the ductal system. This sign might represent ductal infiltration or angiogenesis. It is actually used for breast lesions but we have used this sign for lymph nodes for the first time. For lymph nodes we did not expect long comet tails but small irregularities of the nodes after contrast enhancement was also noted as tail sign.

While some previous studies (Memarsadeghi et al. 2006) have evaluated only the largest lymph node, we especially evaluated nodes where fatty hilum was not observed and comet tail sign was positive. We chose the most suspicious lymph node regardless of its size, as the representative lymph node. MR images of lymph nodes were evaluated by two radiologists, one of which was a breast radiologist. If the results differed then the images were re-evaluated and a consensus was reached. All data obtained from this evaluation was compared with postoperative SLNB results.

### Statistical analyses

Statistical analyses was performed using Number Cruncher Statistical System (NCSS) 2007 (Kaysville, UT, USA). Apart from descriptive statistical methods (average, standard deviation, median, frequency, rate, minimum, maximum) quantitative data was compared using Student’s t test when variables showed normal distribution and Mann–Whitney U test when variables were not distributed normally. Qualitative data was compared using Fisher’s exact test. Yates continuity correction test (Yates correction Chi Square) and diagnostic screening tests were used (specificity, sensitivity etc.). Statistical significance was evaluated at p < 0.01 and p < 0.05.

## Results

Mean age of women was 52.86 ± 13.24. There was no statistical difference between the ages of patients for those with reactive lymph nodes versus those with metastatic nodes (p > 0.05).

Metastatic lymph node was observed as invasive ductal carcinoma metastasis in 85.7 % (n = 30), invasive lobular carcinoma metastasis in 8.6 % (n = 3) and medullary carcinoma metastasis in 5.7 % (n = 2) patients. Lymph nodes were smooth contoured in 85.7 % (n = 30), irregular contoured in 14.3 % (n = 5). Their echogenicity was homogeneous in 85.7 % (n = 30) and heterogenous in 14.3 % (n = 5).

The long axis measurements of lymph nodes ranged from 4 to 28 mm (average 12.37 ± 5.78 mm) while the short axis measurements ranged from 2 to 19 mm (average 6.94 ± 3.62 mm). The long axis to short axis ratio ranged from 1 to 4.6 (average 1.91 ± 0.72). While there was no statistically significant correlation between the long axis measurement and whether the node was reactive or metastatic; metastatic nodes were found to have a longer short axis when compared to reactive nodes (p = 0.042; p < 0.05). The long axis to short axis ratio was notably lower in metastatic nodes when compared to reactive nodes. although this was not statistically significant (p = 0.071; p > 0.05).

Cortical thickness ranged from 1 to 9 mm (average 3.25 ± 2.16 mm) and the cortical thickness to AP diameter ratio ranged from 0.18 to 0.90 (average 0.46 ± 0.17). Cortical thickness was higher in metastatic nodes when compared to reactive nodes (p = 0.024; p < 0.05) (Table 1). There was no statistically significant difference between reactive or metastatic nodes with regards to cortical thickness to AP diameter ratio (p > 0.05).

**Table 1:** Evaluation of pathological status of axillary lymph nodes

	Pathological status		*p*
	Metastatic (n = 15)	Reactive (n = 20)	
Age			
Average ± SD	51.8 ± 14.82	53.65 ± 12.27	0.689^a^
Min–Max	26–80 (49)	35–73 (54)	
Long axis (mm)			
Average ± SD	12.67 ± 5.05	12.15 ± 6.38	0.798^a^
Min–Max	6–22 (12)	4–28 (9.5)	
Short axis (mm)			
Average ± SD	8.00 ± 3.34	6.15 ± 3.70	0.042*^,b^
Min–Max	2–14 (8)	2–19 (5)	
Long/short axis			
Average ± SD	1.69 ± 0.64	2.07 ± 0.76	0.071^b^
Min–Max	1–3 (1.5)	1.3–4.6 (1.8)	
Cortical thickness (mm)			
Average ± SD	4.11 ± 2.30	2.60 ± 1.84	0.024*^,b^
Min–Max	1.2–9 (3)	1–9 (2)	
Cortical thickness/AP diameter			
Average ± SD	0.51 ± 0.21	0.42 ± 0.13	0.167^a^
Min–Max	0.22–0.9 (0.6)	0.18–0.7 (0.41)	

	n (%)	n (%)	
Comet tail sign			
Present	11 (73.3)	1 (5.0)	0.001**^,c^
Not present	4 (26.7)	19 (95.0)	
Fatty hilum			
Not present (obscured)	9 (60.0)	0 (0.0)	0.001**^,d^
Present (intact)	6 (40.0)	20 (100.0)	

* p < 0.05; ** p < 0.01

^a^Student t test

^b^Mann–Whitney U test

^c^Yates continuity correction test

^d^Fisher’s exact test

Comet tail sign was observed in 15 of metastatic nodes (73.3 %) and in one (5 %) reactive node. This difference was statistically significant (p = 0.001; p < 0.01). The Odds ratio was calculated as 52.250 (95 % CI 5.167–528.342). The sensitivity, specificity and accuracy was calculated as 73.33, 95 and 86.71 % respectively. Positive predictive value was 91.67 % and negative predictive value was 82.61 % (Table 2).

**Table 2:** Presence of comet tail sign versus pathological status

	Pathology						p^a^
	Metastatic		Reactive		Total		
	n	%	n	%	n	%	
Comet tail sign							
Present	11	31.4	1	2.9	12	34.3	0.375
Not present	4	11.4	19	54.3	23	65.7	
Total	15	42.9	20	57.1	35	100	
Sensitivity (%)	73.33						
Specificity (%)	95.00						
Positive predictive value (%)	91.67						
Negative predictive value (%)	82.61						
Accuracy (%)	85.71						

^a^McNemar test

While fatty hilum was seen in 40 % of metastatic nodes (n = 6), it was seen in all (n = 20) reactive nodes. This difference was statistically significant (p = 0.001; p < 0.01). The sensitivity specificity and accuracy was 60 %. 100 and 82.86 %, respectively. The positive predictive value was 100 % and negative predictive value was 76.92 % (Table 3).

**Table 3:** Presence of fatty hilum versus pathological status

	Pathology						p^a^
	Metastatic		Reactive		Total		
	n	%	n	%	n	%	
Fatty hilum							
Present (intact)	9	25.7	0	0	9	25.7	0.031*
Not present (obscured)	6	17.1	20	57.1	26	74.3	
Total	15	42.9	20	57.1	35	100.0	
Sensitivity for malignant lymph nodes (%)	60.00						
Specificity for malignant lymph nodes(%)	100.00						
Positive predictive value for malignant lymph nodes(%)	100.00						
Negative predictive value for malignant lymph nodes(%)	76.92						
Accuracy (%) for detecting malignant lymph nodes(%)	82.86						

* p < 0.05

^a^McNemar test

## Discussion

Nowadays, fewer modified radical mastectomies are being planned in the surgical treatment of breast cancer and breast sparing surgery has become the treatment of choice. Similar approaches are taken for the axilla and unnecessary axillar dissections are avoided. No doubt, this change has been brought about by commonly seen complications after axillary dissection, such as pain, edema, neuropathy, long hospital stay and prolonged wound healing.

The axilla, is the most common area of metastasis for breast cancer (Kvistad et al. 2000). The presence of axillary metastasis is important for prognosis and determining the treatment plan. Nowadays, SLNB is used to determine the status of axillary lymph nodes. While a positive SLNB leads to axillary dissection, a negative SLNB saves the patient from further dissection and its related complications (Hyun et al. 2016). However, even this approach can be considered invasive. There are several studies regarding ideal and non-invasive imaging techniques for the detection of axillary lymph node status, such as ultrasound, ultrasound guided fine needle aspiration, PET–CT, contrast-enhanced MRI, ultra-small super-paramagnetic iron oxide (USPIO)-MRI and diffusion-weighted MRI. Due to user dependence, ultrasound guided fine needle aspiration biopsy has a wide sensitivity range, that is reported to be between 25 and 97 % (Bedrosian et al. 2003; Sahoo et al. 2007). Previous studies have noted that while US make the distinction between N0 and N1 for lymph node staging, it cannot distinct between N2 and N3 (Hyun et al. 2016; van Schipper et al. 2013; Caudle et al. 2014). Comparison of PET–CT and MRI have concentrated more on MRI, due to the use of ionizing radiation in PET–CT (Meng et al. 2011). While these methods are unable to detect micrometastasis, micrometastasis are known not to effect survival (Kuijs et al. 2015). In a study by Guiliano et al. on patients with limited axillary metastasis, no significant difference in survival was found between patients undergoing ALND and those not (Giuliano et al. 2011).

Mortorello et al. reported a statistically significant correlation between metastasis on pathology and the presence of a single or multiple nodes with no fatty hilum in contrast enhanced MRI. The same study reported no correlation between pathological lymph node positivity and contrast enhancing kinetics, number of lymph nodes or lymph node sizes (Mortellaro et al. 2009). Our study also found significant correlation between no fatty hilum and metastatic lymph nodes.

Luciani et al. reported that small axis length >4 mm as a predictive value. The presence of cortical irregularities and cortical thickness of >3 mm was found to be statistically significantly correlated with the presence of metastatic lymph nodes (Luciani et al. 2009). When we compared reactive and metastatic nodes, we also found that cortical thickness was higher in metastatic lymph nodes.

It has been reported that metastatic lymph nodes have similar contrast enhancement kinetics with primary breast masses on MRI, and that metastatic lymph nodes demonstrate wash-out similar to malignant lesions (Kvistad et al. 2000). This can be explained through aggressive neoangiogenesis and increased capillary permeability. We, therefore believe that imaging findings of malignant breast masses on postcontrast images may be beneficial in determining the metastatic status of axillary lymph nodes. We evaluated the presence of comet tail sign in metastatic lymph nodes. To our knowledge such a correlation has not been previously investigated. Our study found that of 15 pathologically confirmed metastatic nodes 11 had comet tail sign, while only 1 of 20 reactive nodes showed the same sign. We therefore believe that this may be important for the evaluation of metastatic status of axillary nodes.

A systematic review published in 2015 by Kuijs et al. included 60 studies and no clear number was given for the number of metastatic lymph nodes on axillary MRI (Kuijs et al. 2015). In order for axillary MRI to routinely and safely replace SLNB, each lymph node must be evaluated as metastatic or reactive. Many studies are required for this purpose. When individually evaluating pathological nodes, many specific findings may be required. At this point, we believe that a positive comet tail sign in pathologically confirmed metastatic node is an important and strong finding.

Our study has some limitations. MR evaluations were performed without using an axillary coil, which led to limited evaluation of the axillary anatomy. Also our patient population is relatively small. We did not use diffusion MRI which is also a promising method for the differential diagnosis between metastatic and benign axillary lymph nodes (Fornasa et al. 2012; Yamaguchi et al. 2015; Chung et al. 2014). Our method might be combined with diffusion weighted MRI for better results. Minimally invasive approaches are being preferred for the treatment of breast cancers. MRI is superior to other imaging modalities when evaluating the axilla, and has the highest sensitivity of 84.7 % (Kuijs et al. 2015). Similar studies are required to gather further evidence of specific imaging findings in metastatic lymph nodes.
